# The Late Dr. Elisha Bartlett

**Published:** 1855-08

**Authors:** 


### THE LATE DR. ELISHA BARTLETT.

THAT illness, the end of which has been for years but too clearly foreseen by his friends, has at last brought to a close the earthly career of this great and good man. Dr. Bartlett died at his paternal mansion, in Woonsocket, R. I., on the 19th of July. The intelligence of his death, in the prime of his manhood and in the midst of his usefulness, will fill with sadness thousands of hearts in every part of our country. A teacher of rare popularity, and an author of high reputation, he was a man so gentle, so genial, so kind-hearted that none could know him but to love him. He passed one winter in Louisville; his friends here will never forget that winter—they can never forget that expanded forehead, that most open and winning smile, and that silver voice to the gentle intonations of which so many listened with delight. He was then an invalid, worn by that disease of which he has just died, and the unsteadiness of his gait, the air of languor, the pallid features, betokening too unerringly the hopelessness of its character, heightened the interest felt in him by his friends. But we are not going to write his eulogy, nor attempt a sketch of his life; that task will be perfomed by one who knew him more intimately than the writer of this brief notice.

